# Vitamin D status and obesity markers in older adults: results from West China Health and Aging Trends study

**DOI:** 10.1186/s12877-021-02449-7

**Published:** 2021-10-07

**Authors:** Yunli Zhao, Wanyu Zhao, Qiukui Hao, Meiling Ge, Yan Zhang, Fengjuan Hu, Ying Lu, Lixing Zhou, Xiaolei Liu, Birong Dong

**Affiliations:** 1 The Center of Gerontology and Geriatrics, West China Hospital, Sichuan University, GuoXueXiang 37, Sichuan 610041 Chengdu, China; 2 Department of Geriatrics, West China Hospital, Sichuan University, Guo Xue Xiang, Sichuan Chengdu, China; 3 National Clinical Research Center for Geriatrics and Department of General Practice, State Key Laboratory of Biotherapy, West China Hospital, Sichuan University, and Collaborative Innovation Center of Biotherapy, Chengdu, China; 4 The Center of Gerontology and Geriatrics, West China Hospital, Sichuan University, GuoXueXiang 37, 610041 Chengdu, Sichuan China

**Keywords:** Body mass index, Obesity markers, Older adults, Vitamin D status, Waist-hip ratio

## Abstract

**Backgrounds:**

Vitamin D deficiency and insufficiency in older adults seems to be common, but the prevalence estimates are lacking in West China. Previous studies suggested that low vitamin D status was associated with obesity. However, most of them evaluated obesity based on body mass index (BMI) and there are no studies at present exploring the association between vitamin D status and different obesity markers. The present study aims to investigate the prevalence of low vitamin D status and evaluate the association between the vitamin D status and different obesity markers among older adults in West China.

**Methods:**

Data was based on the baseline of West China Health and Aging Trends study (WCHAT). All of the participants were older than 60 years old in the present study. Vitamin D status was based on laboratory data, and obesity markers were assessed by bioelectrical impedance analysis (BIA) using the InBody 770 analyzer. Multiple linear regression was performed to find the association between the vitamin D status and various obesity markers.

**Results:**

The study included 2661 individuals (mean age: 67.7 ± 6.0 years; males: 41 %). The mean vitamin D level was 18.8 ± 6.3 ng/ml (range: 5 to 59 ng/ml); 5.2 % of participants had a sufficient level of vitamin D, 31.8 % had vitamin D insufficiency, and 63.0 % had vitamin D deficiency. Our results showed that vitamin D status was negatively associated with fat mass index (FMI), visceral fat area (VFA), and waist-hip ratio (WHR) in both sexes. Comparing to other obesity markers, WHR had the strongest correlation with vitamin D status in both sexes (β = -6.090, *P* = 0.046 in males; β = -11.253, *P* < 0.001 in females). No significant association was found between vitamin D status and BMI in males.

**Conclusion:**

The prevalence of vitamin D insufficiency and deficiency among older adults in West China was high. Among the older adults in west China, WHR showed stronger association with vitamin D status and was better for the prediction of vitamin D insufficiency or deficiency in both sexes, compared to BMI.

**Trial registration:**

Chinese Clinical Trial Registry: ChiCTR1800018895.

## Introduction

With the steady increase in longevity over the past several decades, the number of individuals reaching old age is unprecedented and has been predicted to reach approximately 2 billion in 2050 [1]. As society ages, the incidence of chronic diseases increases as well. Therefore, the prevention and treatment of chronic diseases are closely related to the promotion of “healthy aging”.

Vitamin D, including vitamin D2 (ergocalciferol) and vitamin D3 (cholecalciferol), is a multifunctional hormone whose receptor is expressed in 36 types of tissues. Vitamin D is essential not only for musculoskeletal health but also to maintain the function of other systems [2, 3]. Its insufficiency or deficiency is associated with osteoporosis, bone fractures, depression, cardiovascular diseases, cancer, and increased mortality [4]. Although vitamin D can be supplied by cutaneous synthesis on sunlight exposure (90 % of supply) and dietary intake (10 %), its insufficiency or deficiency remains a global health issue affecting more than one billion people, particularly the elderly population [5, 6]. Several factors are associated with vitamin D status, including geographic latitude, altitude, seasons, dietary vitamin D intake, ethnicity, lifestyle and chronic diseases [6–8]. Interestingly, vitamin D status seems to differ upon sex too, with some studies demonstrating that higher vitamin D status in females [9], and others in males [10].

Obesity, another global public health challenge in older adults, is associated with the risk of morbidities, such as cardiovascular disease, type 2 diabetes mellitus, and sleep apnea [11]. Moreover, obese elderly exhibiting physical function decline are more likely to be admitted to a nursing home than their non-obese peers [12]. The increasing prevalence of obesity in older adults is mainly driven by an overall pandemic of obesity in the general population [13]. Many distinct measures, including body mass index (BMI), waist circumstance (WC), waist-hip ratio (WHR), visceral fat area (VFA), and percent body fat (PBF), have been designed to assess the excess adiposity [14]. At present, BMI is the indicator most commonly used to classify obesity. However, an important limitation of BMI is that it cannot reflect the distribution of body fat which is significantly different between males and females [15]. For instance, males have more visceral adipose tissue around the abdomen, creating an “apple shape” distribution, while females have more subcutaneous adipose tissue in the hips and thighs, creating a “pear shape” body habitus [15].

Although a relevant number of epidemiological investigations suggested that low vitamin D status was associated with obesity, only in few instances the associations between vitamin D status and distinct obesity markers were assessed in the same observational study [16, 17]. As most of the studies analyzed the association between vitamin D status and obesity based on BMI, the association between vitamin D status and fat distribution in different parts of the body in older adults remains unclear. Therefore, the aim of our study was to investigate the prevalence of low vitamin D status and evaluate the association between vitamin D status and different obesity markers in the elderly population of West China. Given the sex differences in vitamin D status and adipose tissue storage, we performed our analysis separately in males and females. The main hypothesis was that the prevalence of vitamin D status was high in West China older adults. We also hypothesized that vitamin D status negatively correlated with obesity and there were sex differences between vitamin D status and obesity markers.

## Methods

### Study design and sample selection

Data were based on the baseline of West China Health and Aging Trends study (WCHAT), a cohort study designed to assess the health status and its influencing factors in West China [18]. The study was conducted in 2018 and approved by the Ethics Committee of West China Hospital, Sichuan University (reference: 2017 − 445). The trial was registered on the Chinese Clinical Trial Registry (ChiCTR1800018895). Participants were aged 50 and older from 18 ethnic groups in Sichuan, Yunnan, Guizhou and Xinjiang province. The latitude ranged from 24.7 to 37°N, and the altitude ranged from 500 to 2560 m in the survey location. All participants (or their legal proxies for those who were unable to sign their names) signed written informed consent forms. For all sample size calculations, the formula of N = Z_α_^2^P(1-P)/δ^2^ was used. According to data literature [19–21], the total sample size was estimated that 2582 participants were required with 0.05 significance and considering a 20 % drop-out rate. In the current study, we only included the participants aged 60 and older with relevant data to analyze the associations between vitamin D status and different obesity markers.

### Assessment of characteristics

The following demographic information was collected by trained investigators in face-to-face interview: age, sex, ethnics (Han, Zang, Qiang, others), marital status (married, and single (unmarried/widowed/divorced), smoking history, drinking history, educational level (illiteracy, primary school, high or secondary school, graduate or above), chronic disease and physical activity (regular physical activity or less physical activity in the past month).

### Measurement of blood sample

Fasting blood samples were collected by trained nurses in the morning when the participants arrived at the study center. Blood handling and collection were carried out under strictly standardized conditions. Vitamin D was measured at baseline only, by assessing serum levels of 25(OH)D. Serum 25(OH)D was measured by using the ARCHITECT i2000SR Immunoassay system (Abbott Healthcare Diagnostics; 1 Kallang Place, Singapore) and the intra-assay coefficient of variation (CV) was less than 5 %. Serum vitamin D levels were defined as “sufficient” (25(OH)D ≥ 30 ng/mL), “insufficient” (20 ≤ 25(OH)D < 30 ng/mL), or “deficient” (< 20 ng/mL) according to the Endocrinology Society [22].

### Assessment of obesity markers and physical function

Standing height and body weight was measured without shoes. BMI was calculated by dividing body weight by height squared (kg/m^2^). Fat mass index (FMI) and VFA were measured by bioelectrical impedance analysis (BIA) using an Inbody 770 (BioSpace, Seoul, Korea), which was a convenient method for the measurement of body composition [23]. Triceps skinfold (TSF) and mid-arm circumference (MAC) were taken midway between the olecranon and acromion in the dominant hand with the arm hanging relaxed at the side. WC was measured at the navel level after normal expiration. Hip circumference (HC) was measured at the widest point of the hip. WHR was calculated by WC and HC. Calf circumference (CC) was measured at the thickest of the leg with the participants in the seated position and with their knee flexed 90°. All circumferences were measured in twice, and the average was considered to be the value for each result.

### Statistical analysis

Analyses were conducted using SPSS software, version 24 (IBM Corporation, Chicago, IL, USA). All continuous variables were checked for normality of distribution by using the Kolmogorov-Smirnov test. Means ± standard deviations (SD) were used to summarize the normally distributed variables, while medians and interquartile range (IQR) were used to summarize the non-normally distributed variables. Count and percentage were used to summarize all categorical variables. Difference of groups were tested by ANOVA/Kruskal-Wallis for continuous variables and the chi square test/Fisher’s exact test for categorical variables according to vitamin D status, respectively. Pearson’s/spearman correlation coefficients were computed between serum 25(OH)D concentrations and obesity markers adjusted by sex. Multiple linear regression was conducted to find the relationship between vitamin D status and obesity markers by adjusting socio-demographic variables, (age, sex, ethnicity, marital status, smoking history, drinking history, education level, altitude), number of chronic diseases and physical activity. β and 95 % confidence intervals (CI) were conducted and P < 0.05 was used to determine whether the effect was significant.

## Results

**Fig. 1:**
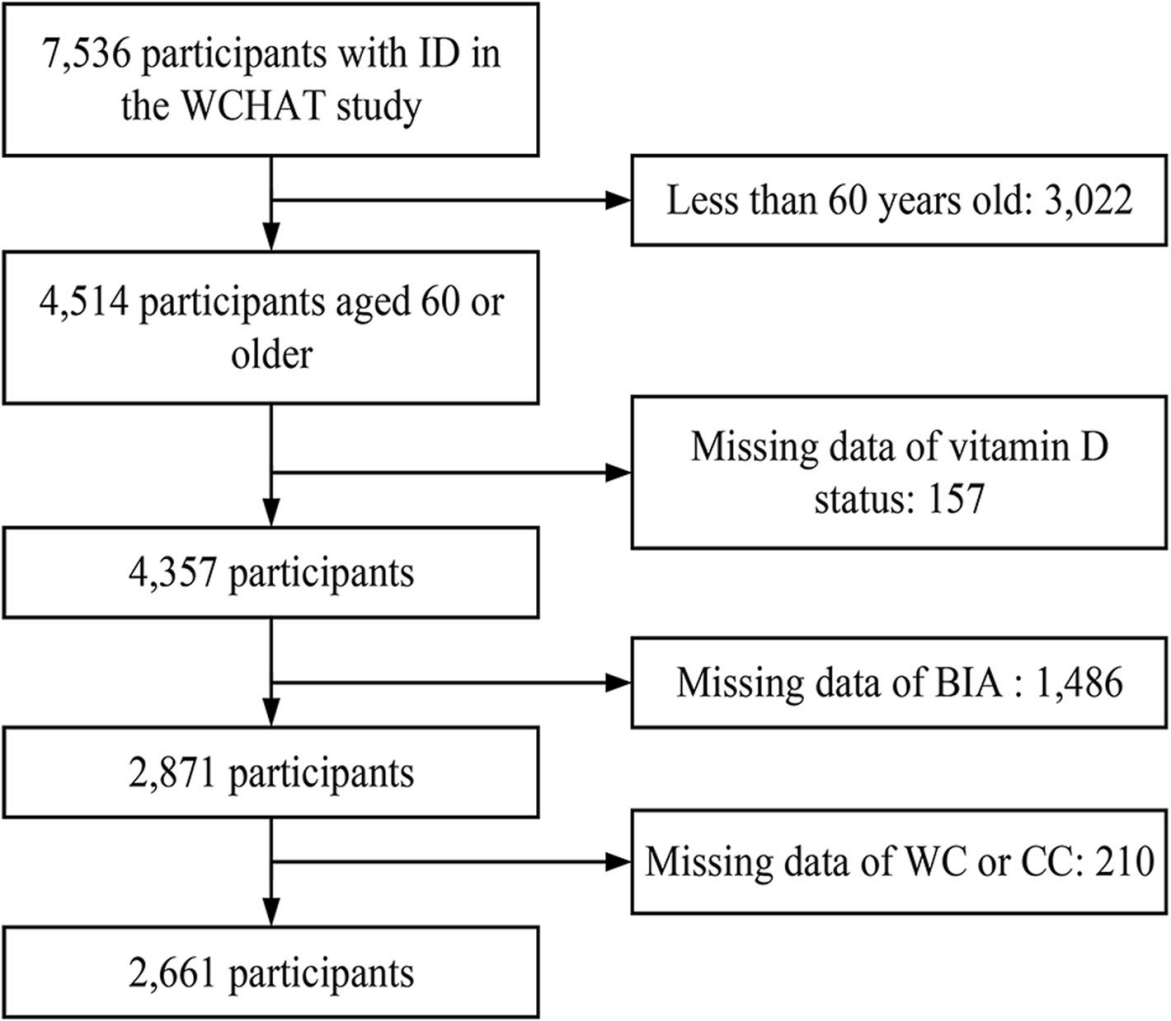
Flow chart of study participants. 7536 participants aged 50 or older from west China were recruited in WCHAT study, 4514 participants aged 60 or older. Among them, 157 participants were excluded because of missing the data of vitamin D status, 1486 participants were excluded because of missing the data of bioelectrical impedance analysis, 210 participants were excluded because of missing the data of WC or CC, 2661 participants were included in our study. *Abbreviations*: *WCHAT* West China Health and Aging Trends study, *BIA* Bioelectrical impedance analysis, *WC* Waist Circumstance, *CC* Calf Circumference

Overall, 2661 participants (41.0 % males) were included in our study (Fig. 1). The mean age of the participants was 67.7 ± 6.0 (range: 60 to 95). The mean vitamin D level was 18.8 ± 6.3 ng/ml (range: 5 to 59 ng/ml). A sufficient vitamin D level was found in 5.2 % of participants, while 31.8 % had vitamin D insufficiency and 63.0 % vitamin D deficiency. Table 1 lists the characteristics of the study participants by the vitamin D status. Age, sex, ethnicity, smoking history, drinking history, education level, marital status, altitude of their place of residence and physical activity were associated with the vitamin D status (P < 0.05).

**Table 1:** Baseline characteristics of the enrolled participants by vitamin D status

Variables		Total (*n* = 2661)	Vitamin D < 20 ng/ml (*n* = 1677)	Vitamin D 20 - 29ng/ml (*n* = 846)	Vitamin D ≥ 30ng/ml (*n* = 138)	*P*-value
Age (years)		67.7 ± 6.0	67.8 ± 6.0	67.0 ± 5.7	67.6 ± 6.5	< 0.001
Gender						
Males (n, %)		1090 (41.0)	526 (31.4)	472 (55.8)	92 (66.7)	< 0.001
Females (n, %)		1571 (59.0)	1151 (68.6)	374 (44.2)	46 (33.3)	
Ethnics (n, %)	Han	1146 (43.1)	641 (38.2)	426 (50.4)	79 (57.2)	< 0.001
	Zang	633 (23.8)	452 (27.0)	152 (18.0)	29 (21)	
	Qiang	696 (26.2)	482 (28.7)	190 (22.5)	24 (17.4)	
	Others	186 (7.0)	102 (6.1)	78 (9.2)	6 (4.3)	
Smoking history (n, %)	Yes	484 (19.2)	237 (15.0)	210 (26.0)	37 (27.8)	< 0.001
Drinking history (n, %)	Yes	677 (26.9)	379 (24.0)	250 (31.0)	48 (36.1)	< 0.001
Educational level (n, %)	Illiteracy	936 (35.2)	648 (38.6)	255 (30.1)	33 (23.9)	< 0.001
	Primary school	1338 (50.3)	788 (47.0)	465 (55.0)	85 (61.6)	
	High or secondary school	208 (7.8)	123 (7.3)	71 (8.4)	14 (10.1)	
	Graduate or above	179 (6.7)	118 (7.0)	55 (6.5)	6 (4.3)	
Marital status (n, %)	Single	649 (24.4)	464 (27.7)	158 (18.7)	27 (19.6)	< 0.001
Altitude (n, %)	0 - 999	394 (14.8)	171 (10.2)	176 (20.8)	47 (34.1)	< 0.001
	1000 - 999	1319 (49.6)	860 (21.3)	416 (49.2)	43 (31.2)	
	≥ 2000	948 (35.6)	646 (38.5)	254 (30.0)	48 (34.8)	
Number of chronic diseases (n, %)	0	1383 (52.0)	74 (48.1)	1235 (52.1)	74 (53.6)	0.444
	1	613 (23.0)	32 (20.8)	552 (23.3)	29 (21.0)	
	≥ 2	665 (25.0)	48 (31.2)	582 (24.6)	35 (25.4)	
Physical activity	Regular physical activity	1893 (75.2)	108 (76.1)	1673 (74.6)	112 (84.2)	0.043
	Less physical activity	625 (24.8)	34 (23.9)	570 (25.4)	21 (15.8)	

Table 2 shows the characteristics of body composition according to the vitamin D status. The mean BMI and VFA of the participants were 25.0 ± 3.7 kg/m^2^ and 105.0 ± 41.3 kg/m^2^, respectively. A higher prevalence of vitamin D deficiency (< 20 ng/ml) was observed in participants with shorter height, higher BMI and FMI, and larger VFA, WC and WHR.

**Table 2:** The characteristics body composition of the enrolled participants by vitamin D status

Variables	Total (*n* = 2661)	Vitamin D < 20 ng/ml (*n* = 1677)	Vitamin D 20–29ng/ml (*n* = 846)	Vitamin D ≥ 30ng/ml (*n* = 138)	*P*-value
Height	155.6 ± 8.3	154.6 ± 8.2	157.0 ± 8.1	158.2 ± 9.0	< 0.001
Weight	60.7 ± 11.0	60.6 ± 11.5	60.9 ± 10.5	60.8 ± 10.0	0.625
BMI (kg/m^2^)	25.0 ± 3.7	25.3 ± 4.1	24.6 ± 3.3	24.2 ± 3.0	< 0.001
FMI (kg/m^2^)	33.4 ± 8.0	34.9 ± 7.9	31.1 ± 7.5	30.1 ± 7.7	< 0.001
TSF	23.0 ± 8.4	23.7 ± 8.3	21.9 ± 8.3	20.4 ± 7.9	< 0.001
VFA	105.0 ± 41.3	111.6 ± 42.7	94.5 ± 36.6	89.7 ± 34.1	< 0.001
MAC	30.4 ± 3.2	30.5 ± 3.3	30.3 ± 3.0	30.2 ± 2.6	0.457
WC	84.1 ± 10.1	84.6 ± 10.5	83.3 ± 9.5	82.7 ± 8.8	0.007
HC	93.9 ± 5.9	94.1 ± 6.1	93.7 ± 5.4	93.6 ± 4.8	0.278
WHR	0.90 ± 0.06	0.90 ± 0.07	0.89 ± 0.06	0.88 ± 0.06	0.001
CC	34.4 ± 3.3	34.3 ± 3.5	34.6 ± 3.1	34.4 ± 2.8	0.271

*Abbreviations*: BMI body mass index, *FMI* fat mass index, *TSF* triceps skinfold thickness, *VFA* visceral fat area, *MAC* mid-arm circumference, *WC* waist circumference, *HC* hip circumference, *WHR* waist hip ratio, *CC* calf circumference

Table 3 shows the correlation between the vitamin D status and body composition in different sexes calculated using Pearson and Spearman correlation coefficients. In males, vitamin D status was negatively correlated with FMI (*r* = -0.102) and WC (*r* = -0.138), while in females, vitamin D status was negatively correlated with weight (*r* = -0.107), BMI (*r* = -0.124), FMI (*r* = -0.139), VFA (*r* = -0.155), AC (*r* = -0.009), WC (*r* = -0.122), HC (*r* = -0.093) and WHR (*r* = -0.122).

**Table 3:** Vitamin D status and body composition of the enrolled participants by sexes

Variables	Correlation(Males)	P	Correlation(Females)	P
Weight	-0.480	0.123	-0.107	< 0.001
Height	-0.043	0.175	0.010	0.706
BMI (kg/m^2^)	-0.028	0.377	-0.124	< 0.001
FMI (kg/m^2^)	-0.102	0.001	-0.139	< 0.001
TSF	-0.072	0.021	0.018	0.491
VFA	-0.138	< 0.001	-0.155	< 0.001
MAC	-0.008	0.781	-0.009	< 0.001
WC	-0.075	0.013	-0.122	< 0.001
HC	-0.038	0.204	-0.093	< 0.001
WHR	-0.009	0.003	-0.122	< 0.001
CC	0.029	0.353	0.003	0.921

*Abbreviations: BMI* body mass index, *FMI* fat mass index, *TSF* triceps skinfold thickness, *VFA* visceral fat area, *MAC* mid-arm circumference, *WC* waist circumference, *HC* hip circumference, *WHR* waist hip ratio, *CC* calf circumference

The relationships between vitamin D status and obesity in the two sexes was further analyzed using the multiple linear regression model (Table 4). In males, FMI (β = -0.155, 95 % CI: -0.308 to -0.003, *P* = 0.046), TSF (β = -0.066, 95 % CI: -0.116 to -0.016, *P* = 0.009), VFA (β = -0.017, 95 % CI: -0.028 to -0.006, *P* = 0.003), and WHR (β = -6.090, 95 % CI: -12.079 to -0.102, *P* = 0.046) were negatively correlated with the vitamin D status. In females, weight (β = -0.58, 95 % CI: -0.89 to -0.028, *P* < 0.001), BMI (β = -0.168, 95 % CI: -0.243 to -0.093, *P* < 0.001), FMI (β = -0.235, 95 % CI: -0.334 to -0.136, *P* < 0.001), VFA (β = -0.018, 95 % CI: -0.025 to -0.011, *P* < 0.001), MAC (β = -0.166, 95 % CI: -0.262 to -0.070, *P* = 0.001), WC (β = -0.070, 95 % CI: -0.102 to -0.039, *P* < 0.001), HC (β = -0.092, 95 % CI: -0.143 to -0.041, *P* < 0.001), WHR (β = -11.253, 95 % CI: -16.390 to -6.116, *P* < 0.001) were negatively correlated with vitamin D status.

**Table 4:** Association between vitamin D status and body composition of the enrolled participants by sexes

Variables	Males			Females		
	β	P	95% CI	β	P	95% CI
Weight	-0.027	0.162	-0.064 - 0.011	-0.58	< 0.001	-0.89 - -0.028
Height	-0.048	0.121	-0.109 - 0.013	0.014	0.601	-0.039 - 0.067
BMI (kg/m2)	-0.034	0.554	-0.148 - 0.080	-0.168	< 0.001	-0.243 - -0.093
FMI (kg/m2)	-0.155	0.046	-0.308 - -0.003	-0.235	< 0.001	-0.334 - -0.136
TSF	-0.066	0.009	-0.116- -0.016	-0.037	0.072	-0.077-0.003
VFA	-0.017	0.003	-0.028 - -0.006	-0.018	< 0.001	-0.025 - -0.011
MAC	-0.001	0.988	-0.131 - 0.129	-0.166	0.001	-0.262 - -0.070
WC	-0.034	0.082	-0.073 - 0.004	-0.070	< 0.001	-0.102 - -0.039
HC	-0.035	0.342	-0.107 - 0.037	-0.092	< 0.001	-0.143 - -0.041
WHR	-6.090	0.046	-12.079 - -0.102	-11.253	< 0.001	-16.390 - -6.116
CC	0.023	0.716	-0.102 - 0.149	-0.033	0.478	-0.123 - 0.058

From multivariate linear regression analyses adjusted for age, sex, ethnicity, marital status, smoking history, drinking history, education level, altitude, number of chronic diseases, and physical activity

*Abbreviations: β* standardized coefficient, *CI* Confidence Interval, *BMI* body mass index, *FMI* fat mass index, *TSF* triceps skinfold thickness, *VFA* visceral fat area, *MAC* mid-arm circumference, *WC* waist circumference, *HC* hip circumference, *WHR* waist hip ratio, *CC* calf circumference

## Discussion

In this study, we found a high prevalence of vitamin D insufficiency (31.8 %) and deficiency (63.0 %) in West China older adults. The prevalence of vitamin D insufficiency and deficiency reported here was higher than most of previous studies performed in older adults in other countries. For example, in the United States, vitamin D insufficiency and deficiency were observed in 41.2 and 27.1 % older adults, respectively [24]. In a European population aged over 80 years, the prevalence of low vitamin D status was 80.9 and 44.5 % when considering cut-offs of 75 and 50 nmol/L [25]. The result of our study reflects a remarkable degree of inadequate vitamin D stores among older adults in West China, as the overall prevalence of 25(OH)D < 75 nmol/l accounted for 94.8 % of the community-dwelling older adults. Therefore, it is essential to develop and implement effective public health strategies to prevent and to control inadequate vitamin D status in West China older adults. Although the exact reason for the high prevalence of vitamin D insufficiency and deficiency in China is uncertain, the low nutritional intake of vitamin D and sun-avoidance behaviors by older adults might be contributing factors [26, 27]. Additionally, racial differences are present among countries, further studies are deserved to confirm whether the use of the same threshold values for determining vitamin D insufficiency or deficiency in different countries is appropriate.

Our results showed that low vitamin D status was associated with obesity in old individuals are consistent with previous studies [28, 29]. The mechanisms linking low vitamin D status and obesity are bi-directional. On the one hand, obese individuals have low sun exposure due to their sedentary lifestyle and diminished outdoor activity. Vitamin D sequestration in adipose tissue, volumetric dilution, vitamin D intake, and diet composition may also contribute to low vitamin D status in obese subjects [30]. On the other hand, many studies supported the possibility that the vitamin D/vitamin D receptor system contributes to the development of obesity by modulating adipocyte differentiation and lipid metabolism [31].

By providing more detailed information, FMI and VFA could better assess total fat mass. In this study, vitamin D status was found to be negatively associated with FMI and VFA in two sexes. Although there are many other methods for measuring FMI and VFA, such as computed tomography (CT), magnetic resonance imaging (MRI), and dual-energy X-ray absorptiometry (DXA), BIA has several advantages including ease of accessibility, no radiation exposure and relatively low cost [8, 32]. Evidence also suggested that Inbody 770 was a reliable tool to measure body fat among ambulatory participants with a delta of − 0.9 ± 2.6 (5 % limits of agreement 6.0 to + 4.2) and a concordance correlation coefficient of 0.97 (95 % CI, 0.96–0.98) when using DXA as a gold-standard measure [32]. However, the validity of Inbody 770 must be interpreted with caution, since it has not been validated in older adults.

Our study also found the correlations of WHR with vitamin D status were stronger than the other obesity markers in both sexes. Several studies demonstrated an inverse correlation between vitamin D status and the risk of abdominal obesity, but most of them included adults of all ages and determined abdominal obesity merely by WC [33]. Few studies had explored the correlation between vitamin D status and WHR. Our finding was consistent with previous literature that WHR was the main predictor of vitamin D levels [34]. One explanation for the finding is that WHR may provide better information on regional fat distribution than other obesity markers. Indeed, increased WHR reflects higher VFA, lower gluteo-femoral fat and muscle mass, while increased WC only suggests higher VFA [35]. Low vitamin D status is associated with both high fat mass and low muscle mass [36]. Thus, the associations between vitamin D status and WHR may be more significant than VFA. Although there are many advantages of WHR over other obesity makers, the measurement of WC and HC cannot be performed or are not reliable in the adults who are unable to stand [37]. Also, due to disc degeneration that results in changes in the form of the spinal column, the measurement of WC is more subject to errors in older people [38], resulting in uncertainty for the association between vitamin D status and these obesity markers. We suggest considering the measurement errors when interpret our results.

In the current study, more obesity markers were found to be related to vitamin D status in females. Weight, BMI, MAC, WC and HC were associated with vitamin D status in females, while only TSF was found in males. The sex differences may reflect the fact that males are more prone to accumulate visceral adipose tissue, while females have greater fat accumulation in subcutaneous (gluteal-femoral) depots than males [39, 40]. Of note, our study did not find the association between vitamin D status and BMI in males. We attributed this lack of correlation to the fact that males have more lean muscle mass than females and the inability of BMI to distinguish between fat mass and lean muscle mass. What’s more, all older adults are at risk of decreasing of muscle mass and increasing of body fat, while BMI could not reflect these changes [41]. These limitations may also suggest that the use of BMI as a marker of obesity may introduce classification errors leading to significant bias when evaluating the risk factors related to obesity.

The active form of vitamin D, 1,25-dihydroxyvitamin D, is thought to play a role in the production of testosterone [42]. Supplemental trials in older males also indicated a significant loss of fat mass in the participants who treated with testosterone [43, 44]. Mechanistically, vitamin D supplementation may be beneficial to fat mass and obesity. However, recent meta-analysis have failed to show a significant decline in the percentage of fat mass [45] or obesity [46] by supplementing vitamin D. The conflicting data from the meta-analysis may be due to heterogeneity in the study populations, the use of different analogues of vitamin D, the frequency (from once a day to once a week) and duration (from 3 to 12 months) of vitamin D supplementation [45], and different outcome measures of regional fat distribution and obesity of the included studies. Therefore, well-designed long term RCTs assessing the effect of vitamin D supplementation in elderly individuals with obesity are needed in the future. Also, recent meta-analysis showed that vitamin D supplementation did not affect physical function, bone fractures, and other health outcomes, the importance of adequate vitamin D status cannot be ignored [47–49]. Vitamin D is not only serving as the cause of illnesses, it could also be the outcome of it. Hence, monitoring the vitamin D level is seems crucial especially among those who has the pivotal predisposition factors.

Several strengths and limitations of our study should be recognized. To our knowledge, this is the first report of the prevalence of vitamin D insufficiency and deficiency and assessed the association between the vitamin D status and several obesity markers in a relatively large sample size of older adults in West China. Second, we used sex-stratified analyses to identify these associations, which only few studies have done before [50]. At the same time, several limitations of this study should be noted. First, similar to other cross-sectional studies, the causal association between vitamin D status and body composition could not be established. Second, the inclusion of only four provinces may lead to inevitable selection bias. Therefore, more provinces should be included in future studies. In addition, we did not measure the parathyroid hormone (PTH) status that might have helped to better explain the association between vitamin D status and obesity. Finally, vitamin D supplementation is a crucial factor that affects vitamin D status among older adults. Unfortunately, we did not investigate the data on vitamin D supplementation in this cohort study, since it was not very common (approximately 0.3 %) among community-dwelling older adults in China [51].

## Conclusions

In summary, the results of this study suggest that the prevalence of low vitamin D status among older adults in West China is high. The inadequate vitamin D status should be a concern for the local government. Importantly, our study compared the associations between different obesity markers and the risk of low vitamin D status. WHR was more strongly associated with low vitamin D status in both sexes than other obesity markers. BMI was not associated with vitamin D status in males. Future studies related to vitamin D status should pay more attention to the older adults with high WHR.

## Data Availability

The datasets used and/or analyzed during the current study available from the corresponding author on reasonable request.

## Abbreviations

BIA: Bioelectrical impedance analysis
BMI: Body mass index
CC: Calf circumference
CI: Confidence intervals
CT: Computed tomography
CV: Coefficient of variation
DXA: Dual-energy X-ray absorptiometry
FMI: Fat mass index
HC: Hip circumference
IQR: Interquartile range
MAC: Mid-arm circumference
MRI: Magnetic resonance imaging
PBF: Percent body fat
RCTs: Randomized controlled trials
SD: Standard deviations
TSF: Triceps skinfold
VFA: Visceral fat area
WC: Waist circumstance
WCHAT: West China Health and Aging Trends study
WHR: Waist-hip ratio

## References

[1] Harper S (2014). Economic and social implications of aging societies. Science.

[2] Chen TC, Chimeh F, Lu Z, Mathieu J, Person KS, Zhang A (2007). Factors that influence the cutaneous synthesis and dietary sources of vitamin D. Arch Biochem Biophys..

[3] Norman AW (2008). From vitamin D to hormone D: fundamentals of the vitamin D endocrine system essential for good health. Am J Clin Nutr..

[4] Autier P, Boniol M, Pizot C, Mullie P (2014). Vitamin D status and ill health: a systematic review. Lancet Diabetes Endocrinol..

[5] Holick MF (2007). Vitamin D deficiency. N Engl J Med.

[6] Cashman KD, Dowling KG, Škrabáková Z, Gonzalez-Gross M, Valtueña J, De Henauw S (2016). Vitamin D deficiency in Europe: pandemic?. Am J Clin Nutr.

[7] Wacker M, Holick MF, Sunlight, Vitamin D (2013). A global perspective for health. Dermatoendocrinol.

[8] Forrest KY, Stuhldreher WL (2011). Prevalence and correlates of vitamin D deficiency in US adults. Nutr Res.

[9] Vallejo MS, Blümel JE, Arteaga E, Aedo S, Tapia V, Araos A (2020). Gender differences in the prevalence of vitamin D deficiency in a southern Latin American country: a pilot study. Climacteric.

[10] Yan X, Zhang N, Cheng S, Wang Z, Qin Y (2019). Gender differences in vitamin D status in China. Med Sci Monit.

[11] Guh DP, Zhang W, Bansback N, Amarsi Z, Birmingham CL, Anis AH (2009). The incidence of co-morbidities related to obesity and overweight: a systematic review and meta-analysis. BMC Public Health..

[12] Marihart CL, Brunt AR, Geraci AA (2015). The high price of obesity in nursing homes. Care Manag J..

[13] Ng M, Fleming T, Robinson M, Thomson B, Graetz N, Margono C (2014). Global, regional, and national prevalence of overweight and obesity in children and adults during 1980–2013: a systematic analysis for the Global Burden of Disease Study 2013. Lancet.

[14] Nimptsch K, Konigorski S, Pischon T (2019). Diagnosis of obesity and use of obesity biomarkers in science and clinical medicine. Metabolism..

[15] Fried SK, Lee MJ, Karastergiou K (2015). Shaping fat distribution: new insights into the molecular determinants of depot- and sex-dependent adipose biology. Obesity (Silver Spring).

[16] Rafiq S, Jeppesen PB (2018). Body mass index, vitamin D. Type 2 diabetes: a systematic review and meta-analysis. Nutrients..

[17] Pereira-Santos M, Costa PR, Assis AM, Santos CA, Santos DB (2015). Obesity and vitamin D deficiency: a systematic review and meta-analysis. Obes Rev..

[18] Hou L, Liu X, Zhang Y, Zhao W, Xia X, Chen X (2021). Cohort profile: West China Health and Aging Trend (WCHAT). J Nutr Health Aging..

[19] Wu C, Smit E, Xue QL, Odden MC (2017). Prevalence and correlates of frailty among community-dwelling Chinese older adults: the China health and retirement longitudinal study. J Gerontol A Biol Sci Med Sci.

[20] Gao L, Jiang J, Yang M, Hao Q, Luo L, Dong B (2015). Prevalence of Sarcopenia and associated factors in Chinese community-dwelling elderly: comparison between rural and urban areas. J Am Med Dir Assoc.

[21] Zhang W, Stoecklin E, Eggersdorfer M (2013). A glimpse of vitamin D status in Mainland China. Nutrition..

[22] Holick MF, Binkley NC, Bischoff-Ferrari HA, Gordon CM, Hanley DA, Heaney RP (2011). Evaluation, treatment, and prevention of vitamin D deficiency: an endocrine society clinical practice guideline. J Clin Endocrinol Metab..

[23] Moro AB, Pires CC, da Silva LP, Dias AMO, Simões RR, Pilecco VM (2019). Prediction of lamb body composition using in vivo bioimpedance analysis. Meat Sci..

[24] Liu X, Baylin A, Levy PD (2018). Vitamin D deficiency and insufficiency among US adults: prevalence, predictors and clinical implications. Br J Nutr..

[25] Bruyère O, Slomian J, Beaudart C, Buckinx F, Cavalier E, Gillain S (2014). Prevalence of vitamin D inadequacy in European women aged over 80 years. Arch Gerontol Geriatr..

[26] Cosman F, de Beur SJ, LeBoff MS, Lewiecki EM, Tanner B, Randall S, et al. Clinician’s guide to prevention and treatment of osteoporosis. Osteoporos Int. 2014;25(10):2359–81. 10.1007/s00198-014-2794-2. 10.1007/s00198-014-2794-2 PMC4176573 25182228

[27] Bouillon R (2017). Comparative analysis of nutritional guidelines for vitamin D. Nat Rev Endocrinol..

[28] Orces CH (2018). The association between obesity and vitamin D status among older adults in Ecuador: analysis of the SABE survey. Nutr Hosp..

[29] Laird E, O’Halloran AM, Carey D, Healy M, O’Connor D, Moore P (2018). The prevalence of vitamin D deficiency and the determinants of 25(OH)D concentration in older Irish adults: data from The Irish Longitudinal Study on Ageing (TILDA). J Gerontol A Biol Sci Med Sci..

[30] Drincic AT, Armas LA, Van Diest EE, Heaney RP (2012). Volumetric dilution, rather than sequestration best explains the low vitamin D status of obesity. Obesity (Silver Spring)..

[31] Savastano S, Barrea L, Savanelli MC, Nappi F, Di Somma C, Orio F (2017). Low vitamin D status and obesity: role of nutritionist. Rev Endocr Metab Disord..

[32] Hurt RT, Ebbert JO, Croghan I, Nanda S, Schroeder DR, Teigen LM (2020). The comparison of segmental multifrequency bioelectrical impedance analysis and dual-energy X-ray absorptiometry for estimating fat free mass and percentage body fat in an ambulatory population. JPEN J Parenter Enteral Nutr.

[33] Hajhashemy Z, Shahdadian F, Ziaei R, Saneei P (2021). Serum vitamin D levels in relation to abdominal obesity: a systematic review and dose-response meta-analysis of epidemiologic studies. Obes Rev..

[34] Abiaka C, Delghandi M, Kaur M, Al-Saleh M (2013). Vitamin d status and anthropometric indices of an omani study population. Sultan Qaboos Univ Med J.

[35] Bouchi R, Asakawa M, Ohara N, Nakano Y, Takeuchi T, Murakami M (2016). Indirect measure of visceral adiposity 'A Body Shape Index’ (ABSI) is associated with arterial stiffness in patients with type 2 diabetes. BMJ Open Diabetes Res Care.

[36] Shantavasinkul PC, Phanachet P, Puchaiwattananon O, Chailurkit LO, Lepananon T, Chanprasertyotin S (2015). Vitamin D status is a determinant of skeletal muscle mass in obesity according to body fat percentage. Nutrition.

[37] Madden AM, Smith S (2016). Body composition and morphological assessment of nutritional status in adults: a review of anthropometric variables. J Hum Nutr Diet.

[38] McAviney J, Roberts C, Sullivan B, Alevras AJ, Graham PL, Brown BT (2020). The prevalence of adult de novo scoliosis: a systematic review and meta-analysis. Eur Spine J.

[39] Karastergiou K, Smith SR, Greenberg AS, Fried SK (2012). Sex differences in human adipose tissues - the biology of pear shape. Biol Sex Differences.

[40] Karpe F, Pinnick KE (2015). Biology of upper-body and lower-body adipose tissued -- link to whole-body phenotypes. Nat Rev Endocrinol.

[41] Cohn SH, Ellis KJ, Yasumura S, Morgan WD (1987). New concepts of body composition. In vivo body composition studies.

[42] Hofer D, Münzker J, Schwetz V, Ulbing M, Hutz K, Stiegler P, Zigeuner R, Pieber TR, Müller H, Obermayer-Pietsch B (2014). Testicular synthesis and vitamin D action. J Clin Endocrinol Metab.

[43] Snyder PJ, Peachey H, Hannoush P, Berlin JA, Loh L, Lenrow DA (1999). Effect of testosterone treatment on body composition and muscle strength in men over 65 years of age. J Clin Endocrinol Metab.

[44] Schroeder ET, Singh A, Bhasin S, Storer TW, Azen C, Davidson T (2003). Effects of an oral androgen on muscle and metabolism in older, community-dwelling men. Am J Physiol Endocrinol Metab.

[45] Golzarand M, Hollis BW, Mirmiran P, Wagner CL, Shab-Bidar S (2018). Vitamin D supplementation and body fat mass: a systematic review and meta-analysis. Eur J Clin Nutr..

[46] Bassatne A, Chakhtoura M, Saad R, Fuleihan GE (2019). Vitamin D supplementation in obesity and during weight loss: a review of randomized controlled trials. Metabolism.

[47] Zhao JG, Zeng XT, Wang J, Liu L (2017). Association between calcium or vitamin D supplementation and fracture incidence in community-dwelling older adults: a systematic review and meta-analysis. JAMA.

[48] Han Q, Li X, Tan Q, Shao J, Yi M (2019). Effects of vitamin D3 supplementation on serum 25(OH)D concentration and strength in athletes: a systematic review and meta-analysis of randomized controlled trials. J Int Soc Sports Nutr..

[49] Zhang Y, Fang F, Tang J, Jia L, Feng Y, Xu P (2020). Association between vitamin D supplementation and mortality: systematic review and meta-analysis. BMJ..

[50] Chiang JM, Stanczyk FZ, Kanaya AM (2018). Vitamin D levels, body composition, and metabolic factors in Asian Indians: results from the metabolic syndrome and atherosclerosis in South Asians living in America pilot study. Ann Nutr Metab..

[51] Chen J, Yun C, He Y, Piao J, Yang L, Yang X (2017). Vitamin D status among the elderly Chinese population: a cross-sectional analysis of the 2010–2013 China national nutrition and health survey (CNNHS). Nutr J.

